# 5-Formylcytosine: a new epigenetic player

**DOI:** 10.1038/s41392-024-02016-7

**Published:** 2024-10-23

**Authors:** Dharmendra Kumar, Iqbal Hyder, Wilfried A. Kues

**Affiliations:** 1 Animal Physiology and Reproduction Division, ICAR-Central Institute for Research on Buffaloes, Hisar, Haryana India; 2 Sri Venkaetswara Veterinary University—NTR College of Veterinary Science, Gannavaram, India; 3 Friedrich-Loeffler-Institut, Federal Research Institute for Animal Health, Biotechnology/Stem Cell Unit, Neustadt, Germany

**Keywords:** Reprogramming, Differentiation, Epigenetics

In a landmark study published in *Cell*, Parasyraki et al. demonstrated a functional role of 5-formylcytosine (5fC) as an epigenetic mark during zygotic/embryonic genome activation (ZGA) in *Xenopus* and murine embryos that is spatially associated with RNA polymerase III (Pol III) transcription.^1^ ZGA (or maternal to embryonic transition) is the unique phase of embryonic development when the maternal genome of the oocyte is reprogrammed to an embryonic gene expression.

ZGA is accompanied by major transcriptional changes, chromatin remodeling, and nuclear reorganization. Among these processes, methylation of cytosine bases (5-methylcytosine, 5mC) is well known for its regulatory role in gene expression in vertebrate genomes. 5mC is an extension of the four-letter genetic code and an important epigenetic mark of eukaryotic genomes associated with numerous biological processes, including zygote formation, embryogenesis, regulation of gene expression, imprinting, and a diverse range of diseases including cancer. During ontogenesis, global demethylation occurs shortly after fertilization, and another wave of cell-specific demethylation to reset DNA imprints during primordial germ cell formation.

Importantly, methylation of cytosines is reversible, and two different mechanisms for demethylation of 5mC have been identified: a passive and an active one. Passive demethylation occurs during DNA replication when the newly synthesized DNA strand is polymerized with unmodified cytosines, resulting initially in hemi-methylated positions; this is thought to occur during the demethylation of DNA in the maternal genome of mouse embryos after fertilization. However, active demethylation is catalyzed by a replication-independent mechanism driven by enzymes of the ten-eleven translocation (TET) dioxygenase family, for example TET dioxygenases oxidize the 5mC in the DNA of paternal mouse pronuclei^2^ in an iterative process to 5-hydroxymethylcytosine (5hmC), 5fC, and 5-carboxylcytosine (5caC). Demethylation is then completed by base excision repair (BER) of the oxidized (oxC)-marks 5fC and 5caC to unmethylated cytosine by thymine DNA glycosylase (TDG).^3^ Earlier, Wossidlo et al. demonstrated that 5hmC promotes transcription factor binding during ZGA, establishing its role in epigenetic reprogramming of the mammalian zygote.^4^ Previous studies have also shown correlations between 5hmC enrichment in enhancers, and 5fC and 5caC in active enhancers and promoters. However, due to the relatively low levels of oxC-marks and possible pleiotrophic effects, it is unclear whether there are causal relationships.^5^

However, the discovery of 5fC as an activator of RNA Pol III-mediated transcription by Parasyraki et al. ^1^ provides a novel perspective on how non-coding RNA transcription is regulated during ZGA in *Xenopus* embryogenesis. Employing a combination of cutting-edge epigenomic profiling, chromatin immunoprecipitation (ChIP)-seq, and functional genomics approaches, it was demonstrated that 5fC is not simply a by-product of DNA demethylation, but rather showed a biphasic profile with a steep, singular increase around ZGA, followed by a slower increase now paralleled by the levels of the other oxC-marks (hmC and hcaC) at later stages of development. Current work demonstrated that 5fC plays an active role in gene regulation during the early stages of development in *Xenopus*.^1^ Through experiments conducted on *Xenopu*s embryos, the researchers reveal the formation of 5fC-rich nuclear chromocenters, particularly at the perinucleolar compartment, a structure critical for RNA Pol III transcription.

**Fig. 1:**
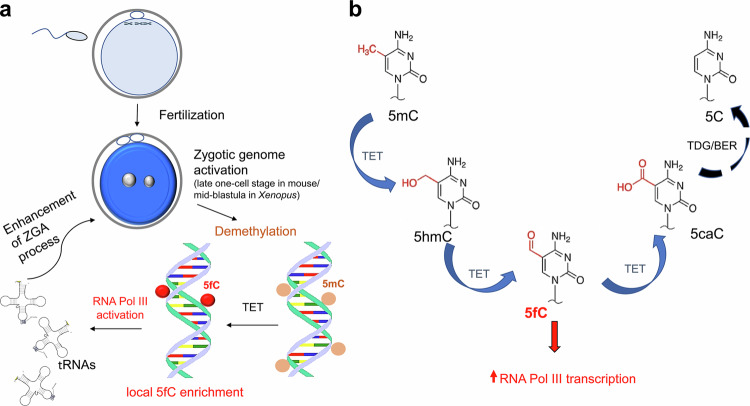
Active demethylation of 5mC by TET dioxygenases and local enrichment of 5fC. **a** 5-Formylcytosine (5fC) acts as epigenetic player specifically recruiting and activating RNA Pol III transcription during ZGA. Note, ZGA occurs at species-specific time points, in mouse embryos at late one-cell stage, in *Xenopus* at mid-blastula. **b** Default model of TET dioxygenase/TDG-catalyzed demethylation of 5mC. However, high-resolution liquid chromatography-tandem mass spectrometry data from *Xenopus* embryos revealed a biphasic profile of 5fC independent of 5mC and 5hmC, and immunohistology indicated specific enrichment of 5fC at chromocenters and active tRNA repeats,^1^ suggesting a specific pathway of 5fC generation or local stabilization

Notably, 5fC was shown to be highly enriched at RNA Pol III target genes, especially on oocyte-specific tandemly arrayed tRNA genes, which are activated during ZGA and are essential for protein synthesis and cellular function. To assess whether 5fC acts as a regulatory epigenetic mark, the researchers knocked down key enzymes involved in DNA demethylation, such as TET 2/3, in addition, the levels of 5fC were artificially reduced by injecting mRNA coding for TDG. Both experimental approaches led to reduced 5fC levels and diminished RNA Pol III transcription. To directly assess the role of 5fC levels in tRNA transcription, plasmids carrying different oxC-marks on a critical cytosine of an artificial tRNA-iMet (initiator methionine tRNA = rate limiting tRNA for cell growth) plasmid were injected into *Xenopus* oocytes, only the 5fC carrying plasmids enhanced RNA Pol III-mediated transcription, confirming its active role in gene regulation. Importantly, the 5fC stimulated expression was most pronounced after injection into the animal pole, suggesting that the 5fC-mediated transcription of tRNA genes is region and stage specific in *Xenopus* embryos. The immunohistological analysis of 5mC and the oxC-marks in murine zygotes, confirmed the transient 5fC enrichment in chromocenters of the male pronucleus suggesting an evolutionary conserved process (Fig. 1).

Mechanistically, the study proposes that 5fC promotes RNA Pol III transcription by increasing chromatin accessibility, as evidenced by ATAC-seq results showing that 5fC-enriched regions exhibit greater openness and disrupted nucleosome positioning. This enhances recruitment of RNA Pol III and associated transcription factors. The identification of 5fC as an activator of RNA Pol III suggests its critical role in ensuring adequate supplies of tRNAs and rRNAs during early development, raising the possibility that mis-regulation of 5fC could impair ZGA and embryonic viability. Several questions remain, including how 5fC is specifically deposited at Pol III-transcribed loci, its interaction with other epigenetic marks, and whether its role is conserved across species, including humans. The apparent local enrichment of 5fC at Pol III loci suggests a specific mechanism for production of this oxC-mark, potentially via interaction with other epigenetic marks, e.g., on nucleosomes. Elucidation of this aspect will be of great interest and warrants further research. The factors required for 5fC recognition and activation of RNA Pol III have not yet been identified. Interestingly, the electrophilic property of the formyl group leads to a weaker hydrogen bonding with the pairing guanosine nucleotide and may contribute to RNA Pol III activation. The data from murine zygotes suggest a conserved mechanism in mouse embryogenesis, but this needs to be validated. It would be fascinating to assess the potential applications of this discovery in assisted reproductive technologies, where modulating 5fC levels could enhance the success of in vitro fertilization (IVF) or somatic cell nuclear transfer (SCNT). Overall, identification of 5fC as an essential epigenetic mark provides new insights into the dynamic regulation of RNA Pol III genes and highlights its critical role in the reprogramming processes necessary for early development. This discovery may have broader implications for understanding epigenetic regulation in other biological contexts and may open new directions for investigating cell commitment, aging, and cancer development and progression.
